# The CD3 versus CD7 Plot in Multicolor Flow Cytometry Reflects Progression of Disease Stage in Patients Infected with HTLV-I

**DOI:** 10.1371/journal.pone.0053728

**Published:** 2013-01-22

**Authors:** Seiichiro Kobayashi, Yamin Tian, Nobuhiro Ohno, Koichiro Yuji, Tomohiro Ishigaki, Masamichi Isobe, Mayuko Tsuda, Naoki Oyaizu, Eri Watanabe, Nobukazu Watanabe, Kenzaburo Tani, Arinobu Tojo, Kaoru Uchimaru

**Affiliations:** 1 Division of Molecular Therapy, Institute of Medical Science, The University of Tokyo, Tokyo, Japan; 2 Department of Molecular Genetics, Medical Institute of Bioregulation, Kyushu University, Fukuoka, Japan; 3 Department of Hematology/Oncology, Research Hospital, Institute of Medical Science, The University of Tokyo, Tokyo, Japan; 4 Laboratory of Diagnostic Medicine, Division of Stem Cell Therapy, Institute of Medical Science, The University of Tokyo, Tokyo, Japan; 5 Clinical Laboratory, Research Hospital, Institute of Medical Science, The University of Tokyo, Tokyo, Japan; Institut Pasteur, France

## Abstract

**Purpose:**

In a recent study to purify adult T-cell leukemia-lymphoma (ATL) cells from acute-type patients by flow cytometry, three subpopulations were observed in a CD3 versus CD7 plot (H: CD3^high^CD7^high^; D: CD3^dim^CD7^dim^; L: CD3^dim^CD7^low^). The majority of leukemia cells were enriched in the L subpopulation and the same clone was included in the D and L subpopulations, suggesting clonal evolution. In this study, we analyzed patients with indolent-type ATL and human T-cell leukemia virus type I (HTLV-I) asymptomatic carriers (ACs) to see whether the CD3 versus CD7 profile reflected progression in the properties of HTLV-I-infected cells.

**Experimental Design:**

Using peripheral blood mononuclear cells from patient samples, we performed multi-color flow cytometry. Cells that underwent fluorescence-activated cell sorting were subjected to molecular analyses, including inverse long PCR.

**Results:**

In the D(%) versus L(%) plot, patient data could largely be categorized into three groups (Group 1: AC; Group 2: smoldering- and chronic-type ATL; and Group 3: acute-type ATL). Some exceptions, however, were noted (*e.g.*, ACs in Group 2). In the follow-up of some patients, clinical disease progression correlated well with the CD3 versus CD7 profile. In clonality analysis, we clearly detected a major clone in the D and L subpopulations in ATL cases and, intriguingly, in some ACs in Group 2.

**Conclusion:**

We propose that the CD3 versus CD7 plot reflects progression of disease stage in patients infected with HTLV-I. The CD3 versus CD7 profile will be a new indicator, along with high proviral load, for HTLV-I ACs in forecasting disease progression.

## Introduction

Human T-cell leukemia virus type I (HTLV-I) is the agent that causes HTLV-I-associated diseases, such as adult T-cell leukemia-lymphoma (ATL), HTLV-l-associated myelopathy/tropical spastic paraparesis (HAM/TSP), and HTLV-I uveitis (HU) [1]–[3]. Approximately 10–20 million people are infected with the HTLV-I virus worldwide [4]. The lifetime risk of developing ATL is estimated to be approximately 2.5–5% [5], [6]. ATL includes a spectrum of diseases that are referred to as smoldering-, chronic-, lymphoma-, and acute-type [7], [8]. The chronic and smoldering types of ATL are considered indolent and are usually managed with watchful waiting until the disease progresses to aggressive (lymphoma- or acute-type) ATL [9]. Because the prognosis of ATL is poor with current treatment strategies, factors to forecast progression to ATL from asymptomatic carriers (ACs) have been researched [10]–[13] in the hope that they will be useful for preventive therapy under development in the early malignant stage.

Various cellular dysfunctions induced by viral genes (*e.g.*, tax and HBZ), genetic and epigenetic alterations, and the host immune system are considered to cooperatively contribute to leukemogenesis in ATL [14]–[16]. However, the complex mechanism may hinder determination of a clear mechanism of the pathology and make discovery of risk factors difficult. In a prospective nationwide study in Japan, high proviral load (VL, over 4.17 copies/100 peripheral blood mononuclear cells) was found to be a major risk factor for HTLV-I AC developing into ATL [13]. Although VL indicates the proportion of HTLV-I-infected cells, it does not indicate size or degree of malignant progression in each clone; *i.e.*, it does not directly indicate progression of disease stage in HTLV-I infection. Moreover, the majority of ACs with high VL remained intact during the study period, indicating that a more accurate indicator of progression is needed.

In our recent study to purify monoclonal ATL cells from acute-type patients by flow cytometry, three subpopulations were observed in a CD3 versus CD7 plot of CD4^+^ cells (H: CD3^high^CD7^high^, D: CD3^dim^CD7^dim^, L: CD3^dim^CD7^low^), and the majority of ATL cells were enriched in the L subpopulation [17]. Clonality analyses revealed that the D and L subpopulations contained the same clone, suggesting clonal evolution of HTLV-I-infected cells to ATL cells. From these findings, we speculated that the CD3 versus CD7 profile may reflect disease progression in HTLV-I infection. In this study, the CD3 versus CD7 profile by flow cytometry, combined with molecular (clonality and proviral load) characterizations, were analyzed in patients with various clinical subtypes (HTLV-I AC, and indolent and aggressive ATL). We found that the CD3 versus CD7 profile reflected disease progression of HTLV-I-infected cells to ATL cells. We also discuss the significance of this analysis as a novel risk indicator for HTLV-I ACs in forecasting progression to ATL.

## Materials and Methods

### Cell lines and patient samples

TL-Om1, an HTLV-I-infected cell line, established Dr. Hinuma's laboratory [18], was provided by Dr. Toshiki Watanabe (The University of Tokyo, Tokyo, Japan) and was cultured in RPMI-1640 medium containing 10% fetal bovine serum. Peripheral blood samples were collected from inpatients and outpatients at our hospital from August 2009 to November 2011. All patients with ATL were categorized according to Shimoyama's criteria [7], [8]. Patients with various complications, such as autoimmune disorder and systemic infections, were excluded. Lymphoma-type patients were excluded because ATL cells are not considered to exist in peripheral blood of this clinical subtype. In patients with ATL receiving chemotherapy, blood samples were collected before treatment or during the recovery phase between chemotherapy sessions. Samples collected from 10 healthy volunteers (mean age: 47.4 years; range: 27–66 years) were used as normal controls.

The present study was approved by the research ethics committee of the institute of medical science, the university of Tokyo. Subjects provided written informed consent.

### Flow cytometry and cell sorting

Peripheral blood mononuclear cells (PBMCs) were isolated from heparin-treated whole blood by density gradient centrifugation, as described previously [17]. Cells were stained using a combination of phycoerythrin (PE)-CD7, APC-Cy7-CD3, Pacific Blue-CD4, and Pacific Orange-CD14. Pacific Orange-CD14 was purchased from Caltag-Invitrogen (Carlsbad, CA). All other antibodies were obtained from BD BioSciences (San Jose, CA). Propidium iodide (PI; Sigma, St. Louis, MO) was added to the samples to stain dead cells immediately prior to flow cytometry. A BD FACS Aria instrument (BD Immunocytometry Systems, San Jose, CA) was used for all multicolor flow cytometry and cell sorting. Data were analyzed using the FlowJo software (Treestar, San Carlos, CA).

### Quantification of HTLV-I proviral load by real-time quantitative polymerase chain reaction (PCR)

The HTLV-I proviral load in FACS-sorted PBMCs was quantified by real-time quantitative polymerase chain reaction (PCR; TaqMan method) using the ABI Prism 7000 sequence detection system (Applied Biosystems, Foster City, CA) as described previously [13], [17]. Briefly, 50 ng of genomic DNA was extracted from human PBMCs using a QIAamp DNA blood Micro kit (Qiagen, Hilden, Germany). Triplicate samples of the DNA were amplified. Each PCR mixture, containing an HTLV-I pX region-specific primer pair at 0.1 µM (forward primer 5′-CGGATACCCAGTCTACGTGTT-3′ and reverse primer 5′-CAGTAGGGCGTGACGATGTA-3′), FAM-labeled probe at 0.1 µM (5′- CTGTGTACAAGGCGACTGGTGCC-3′), and 1× TaqMan Universal PCR master mix (Applied Biosystems), was subjected to 50 cycles of denaturation (95°C, 15 seconds) and annealing to extension (60°C, 1 minute), following an initial Taq polymerase activation step (95°C, 10 minutes). The RNase P control reagent (Applied Biosystems) was used as an internal control for calculating the input cell number (using VIC reporter dye). DNAs extracted from TL-Om1 and normal human PBMCs were used as positive and negative controls, respectively. The HTLV-I proviral load (%) was calculated as the copy number of the pX region per input cell number. To correct the deviation of data acquired in each experiment, data from TL-Om1 (positive control) were adjusted to 100%, and the sample data were corrected accordingly by a proportional calculation.

### Inverse long PCR

For clonality analysis, inverse long PCR was performed [17]. First, 1 µg of genomic DNA extracted from the FACS-sorted cells was digested with *Eco*RI and *Pst*I at 37°C overnight. Purification of DNA fragments was performed using a QIAEX2 gel extraction kit (Qiagen). The purified DNA was self-ligated with T4 DNA ligase (Takara Bio, Otsu, Japan) at 16°C overnight. The circular DNA obtained from the *Eco*RI digestion fragment was then digested with *Mlu*I, which cuts the pX region of the HTLV-I genome and prevents amplification of the viral genome. Inverse long PCR was performed using Takara LA *Taq* polymerase (Takara Bio). For the *Eco*RI-treated template, the forward primer was 5′-TGCCTGACCCTGCTTGCTCAACTCTACGTCTTTG-3′ and the reverse primer was 5′-AGTCTGGGCCCTGACCTTTTCAGACTTCTGTTTC-3′. For the *Pst*I-treated group, the forward primer was 5′-CAGCCCATTCTATAGCACTCTCCAGGAGAG-3′ and the reverse primer was 5′-CAGTCTCCAAACACGTAGACTGGGTATCCG-3. Each 50-µL reaction mixture contained 0.4 mM of each dNTP, 25 mM MgCl_2_, 10× LA PCR buffer II containing 20 mM Tris-HCl and 100 mM KCl, 0.5 mM of each primer, 2.5 U LA *Taq* polymerase, and 50 ng of the processed genomic DNA. The reaction mixture was subjected to 35 cycles of denaturation (94°C, 30 seconds) and annealing to extension (68°C, 8 minutes). Following PCR, the products were subjected to electrophoresis on 0.8% agarose gels. In samples from which a sufficient amount of DNA was extracted, PCRs were performed in duplicate.

## Results

### CD3 versus CD7 profile in flow cytometry in various clinical subtypes of patients infected with HTLV-I

**Figure 1:**
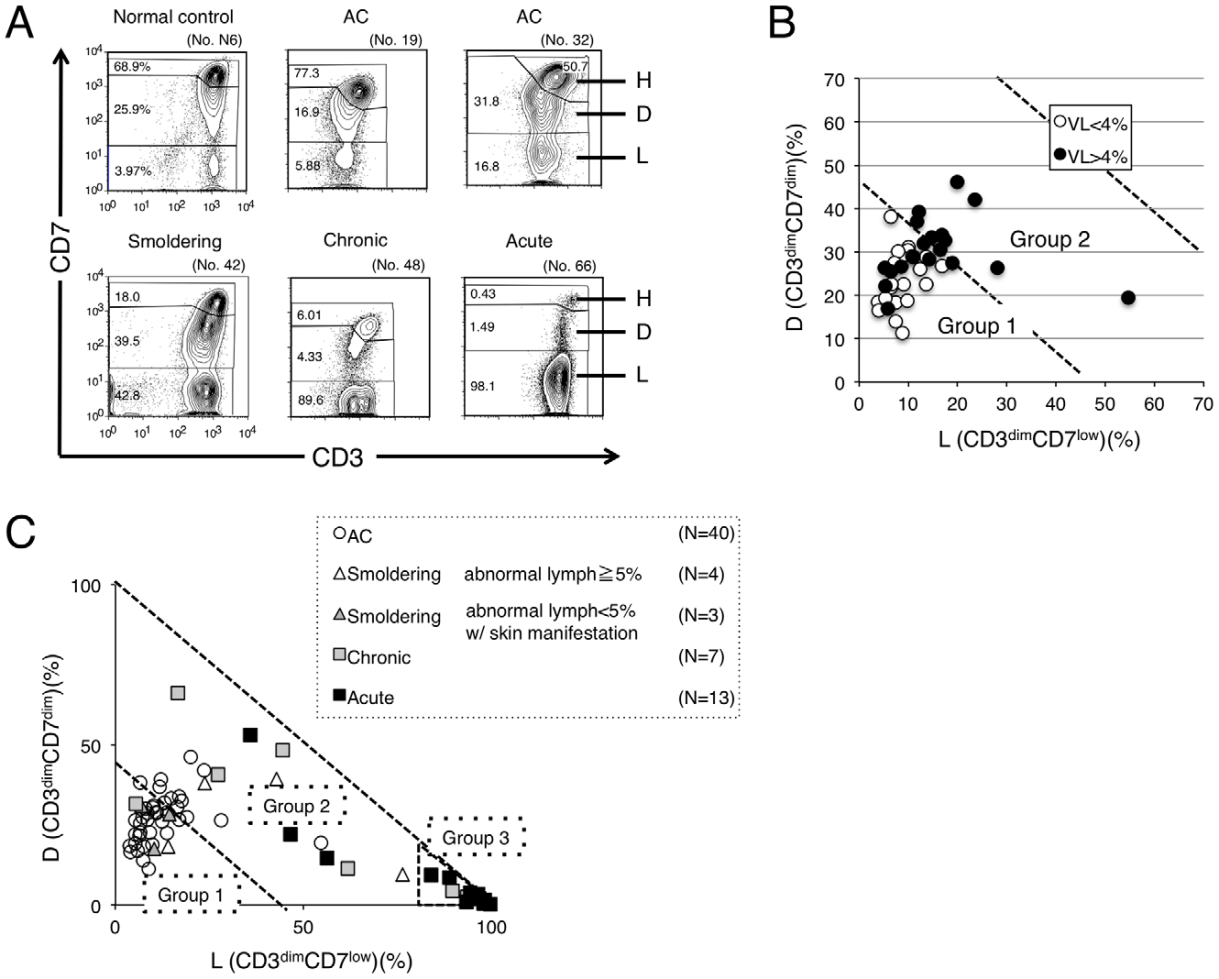
CD3 versus CD7 plots in flow cytometric analysis of patients who are asymptomatic HTLV-I carriers (ACs) and have various clinical subtypes of adult T-cell leukemia-lymphoma (ATL) suggest disease progression in HTLV-I infection. (A) Flow cytometric profile of an AC, various clinical subtypes of ATL (smoldering, chronic, and acute), and a normal control. Representative cases of CD3 versus CD7 plots in CD4^+^ cells are shown. (B) A two-dimensional plot of AC cases showing the percentage of the D and L subpopulations by flow cytometry. AC cases were divided into two groups according to HTLV-I VL (greater or less than 4%). The border line (45% of D+L subpopulations) between Group 1 and 2 was set based on proviral load (VL). All AC cases with less than 4% VL were included in Group 1. All AC cases included in Group 2 had greater than 4% VL. VL<4%: n = 21; VL>4%: n = 19. All VL data in this figure were provided from the database of the Joint Study on Predisposing Factors of ATL Development (JSPFAD). (C) A two-dimensional plot of all patients showing the percentage of the D and L subpopulations. The smoldering type was divided into two categories: smoldering type with greater than 5% abnormal lymphocytes and smoldering type with less than 5% abnormal lymphocytes with skin manifestation. The two diagonal dotted lines indicate 45% and 100% of D+L subpopulations (*i.e.*, 55% and 0% of the H subpopulation). Data were categorized into three groups.

The clinical profiles of the 77 cases analyzed in this study are shown in Table 1. According to the gating procedure, as shown in Figure S1 [17], we constructed a CD3 versus CD7 plot of CD4^+^ cells in PBMCs of various clinical subtypes from patients infected with HTLV-I and normal controls. The three subpopulations (CD3^high^CD7^high^, CD3^dim^CD7^dim^, and CD3^dim^CD7^low^) observed are referred to as the H, D, and L subpopulations, respectively. Representative results for each clinical subtype of HTLV-I infection are shown in Figure 1A. Regarding the data for an acute-type patient (no. 66), the dominant population was the L subpopulation, in which we previously demonstrated that monoclonal ATL cells are enriched [17]. Regarding the AC (no. 19), the CD3 versus CD7 profile was close to that of the normal control, although in some AC cases, such as no. 32, the profile differed from that of the normal control, because in contrast to case no. 19, these cases had increased D and L subpopulations. Regarding the data for indolent-type disease (smoldering and chronic), increases in the D and L subpopulations were intermediate between ACs and patients with acute-type disease. These representative flow cytometric data suggest that continuity in the CD3 versus CD7 profile seemed to exist among the various clinical subtypes of patients infected with HTLV-I.

**Table 1:** Clinical profile of patients infected with HTLV-I and normal controls.

Clinical sutype	Number	Male	Female	Age	WBC(/µl)	Lymphocytes(%)	Abnormal lymphocytes(%)
	of cases			(range)	(range)	(range)	(range)
HTLV-1 AC	40	12	28	49.9	5525	35.9	0.9
				(28–70)	(2680–10360)	(22.4–59.5)	(0.0–4.4)
Smoldering	7	4	3	55.3	5944	32.5	5.8
				(43–77)	(3680–8710)	(13.4–47.5)	(0.7–16.5)
Chronic	7	4	3	52.7	9180	45.8	9.2
				(37–60)	(4070–12790)	(35.0–61.5)	(3.4–12.7)
Acute	13	4	9	58.8	15328	16.3	40.3
				(42–74)	(4450–41480)	(1.7–50.5)	(3.0–89.6)
Normal controls	10	6	4	47.4	ND	ND	ND
				(27–66)			

WBC: white blood cells (normal range, 3500–9100/µl).

AC: asymptomatic carrier.

ND: analysis were not performed.

Average of age, WBC, lymphocytes (%) and abnormal lymphocytes (%) are shown.

The proportion of abonormal lymphocytes in peripheral blood WBCs was evaluated by morphological exmanination.

The proportions of D and L subpopulations in all AC cases analyzed are shown in Figure 1B. Because the high HTLV-I proviral load (VL) in whole PBMCs, a VL of >4%, was reported to be a major risk indicator for progression to ATL [13], a border line was set based on VL. Group 1, the area under the diagonal line (D+L = 45%), included all AC cases with VLs of <4%. ACs with VLs of >4% were distributed between Groups 1 and 2. The proportions of D and L subpopulations in normal controls are shown in Figure S2. In this plot, all data for normal controls were distributed in Group 1. Data for all clinical subtypes are shown in Figure 1C. Most data for acute-type patients were located in the area beyond 80% of the L subpopulation and we designated this area as Group 3. Group 2, which is located between Group 1 and Group 3, included the majority of indolent-type (smoldering and chronic) cases. From these results, the three groups in the D(%) versus L(%) plot seemed to represent disease stage in each case.

### Proviral load and clonality in each subpopulation in the CD3 versus CD7 plot

**Figure 2:**
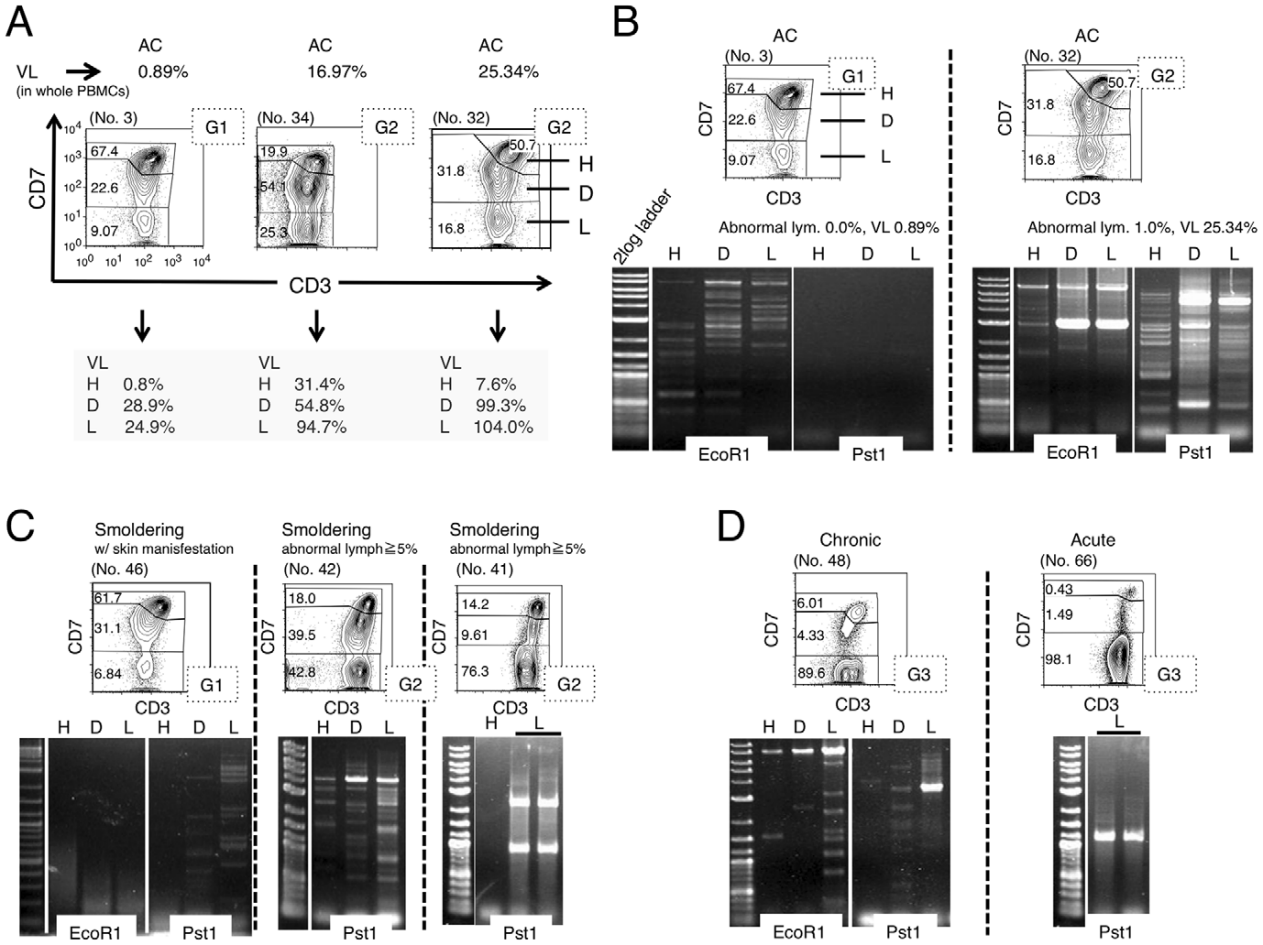
HTLV-I proviral load (VL) and clonality in each subpopulation, based on the CD3 versus CD7 plot. (A) The three subpopulations (H, D, L) based on the CD3 versus CD7 plot were subjected to fluorescence-activated cell sorting (FACS) and VL analysis. Three representative cases are shown. G1 or G2 in the dotted box indicates Group 1 or Group 2, categorized by the percentage of the D and L subpopulations, respectively. (B)–(D) Analysis of clonality in the three subpopulations based on the CD3 versus CD7 plot. Genomic DNA was extracted from FACS-sorted cells of each subpopulation and subjected to inverse long polymerase chain reaction (PCR). Representative data of two cases of AC (B), three cases of smoldering type, including one with skin manifestations (C), and cases of a chronic type and an acute type (D) are shown. PCR was performed in duplicate (black bars) in cases when a sufficient amount of DNA was obtained.

To further characterize each subpopulation (H, D, and L) in the CD3 versus CD7 plot, cells in each subpopulation were FACS-sorted and subjected to analysis of VL to determine the percentage of HTLV-I-infected cells in each subpopulation. Results for representative cases are shown in Figure 2A. The VL in whole PBMCs of an AC (no. 3) was low (0.89%). As expected, the VL in H, the major subpopulation, was low (0.8%). However, VLs in the D and L subpopulations were considerably higher (28.9% and 24.9%, respectively), indicating that HTLV-I-infected cells are relatively concentrated in these subpopulations. In the cases with high VLs in whole PBMCs (no. 32 with 25.34%; no. 34 with 16.97%), the VLs were also higher in the D and L subpopulations, and almost all cells in the L subpopulation were HTLV-I-infected.

In HTLV-I infection, progression to ATL requires several pathological steps, including clonal expansion [15]. To further characterize the three subpopulations based on the CD3 versus CD7 plot, we analyzed clonality in each subpopulation in patients with various clinical subtypes using the inverse long PCR method. Figure 2B shows two cases of AC. In the left case (no. 3), included in Group 1 in the D(%) and L(%) plot, multiple bands suggestive of multiple small clones were detected in the three subpopulations. However, no major band suggestive of a dominant clone was observed. In the right case (no. 32), included in Group 2, inverse long PCR of the FACS-sorted subpopulations suggested that the D and L subpopulations contained a major clone (Figure 2B). The D subpopulation had bands of the same size as those of the L subpopulation, indicating that the two distinct subpopulations contained a common major clone. Eleven cases of AC were included in Group 2. All three cases analyzed by Southern blotting (whole blood samples) were positive for clonal bands (Figure S3). In Figure 2C, data for three smoldering cases are shown. In case no. 46 (left), whose only manifestation was a skin eruption with few abnormal lymphocytes (less than 5% of white blood cells) in the peripheral blood, only faint minor bands suggestive of small clones were observed. In contrast, in the other two cases (nos. 42 and 41), intense bands suggestive of major clones were observed in both the D and L subpopulations. In no. 41 (right), weak bands were not visible, which suggested the selection of dominant clones. In Figure 2D, data for a chronic-type case and an acute-type case are shown. In both cases, intense bands in the L subpopulation suggest the existence of a major clone. The series of clonality analyses indicated that a major clone became more evident and the clinical stage became more advanced as the D and L subpopulations increased.

### Clinical evaluation of exceptional cases categorized by proportions of the CD3^dim^CD7^dim^ (D) and CD3^dim^CD7^low^ (L) subpopulations

**Figure 3:**
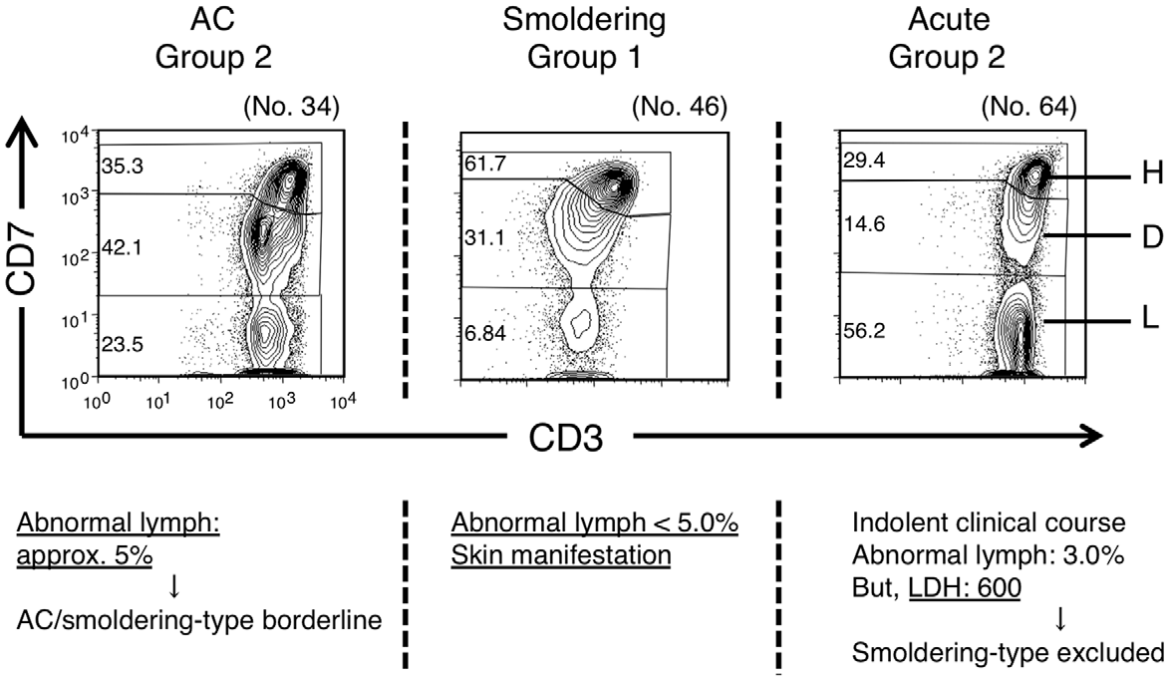
Study of exceptional cases categorized by proportion of the CD3^dim^CD7^dim^ (D) and CD3^dim^CD7^low^ (L) subpopulations. Left: An HTLV-I AC patient who was categorized in Group 2 in the D(%) versus L(%) plot. Middle: A patient with smoldering-type ATL who was categorized in Group 1. Right: A patient with acute-type ATL who was categorized in Group 2.

As noted above, the D(%) versus L(%) plot generally represented disease stage in HTLV-I infection. However, we observed one case of chronic-type disease and three cases of smoldering-type disease in Group 1 and three cases of acute-type disease in Group 2. Furthermore, some ACs with VLs of >4% were observed in Group 2. Representative data from these apparently exceptional cases are shown in Figure 3. On the left, a case of AC (no. 34) observed in Group 2 is shown. 4.7% of lymphocytes in this blood sample were abnormal and clonality analysis by Southern blotting showed oligoclonal bands suggestive of clones of substantial size (Figure S3). These clinical data suggest that the disease stage would be around the AC/smoldering borderline. In the middle, a case of a smoldering type (no. 46) observed in Group 1 is shown. In this case, the percentage of abnormal lymphocytes in the peripheral blood was only 1%, but she had a histologically proven ATL lesion in the skin and was diagnosed with smoldering-type ATL. The other two smoldering cases categorized in Group 1 were the same as this case. These results indicate that ATL cells in these three smoldering cases infiltrated the skin, but not the peripheral blood. On the right, a case of acute-type disease categorized as Group 2 (no. 64) is shown. The clinical course of this patient was relatively indolent compared with typical acute-type disease. He had skin infiltration of ATL cells, but no lymph node swelling. However, LDH exceeded 1.5 times the upper limit of the normal range, which excludes a diagnosis of smoldering-type disease. Other acute-type cases categorized in Group 2 were diagnosed as such according to Shimoyama's criteria, but also had the same indolent clinical course as case no. 64. These cases should have been regarded as indolent ATL.

### Changes in the CD3 versus CD7 profile in flow cytometry with disease progression

**Figure 4:**
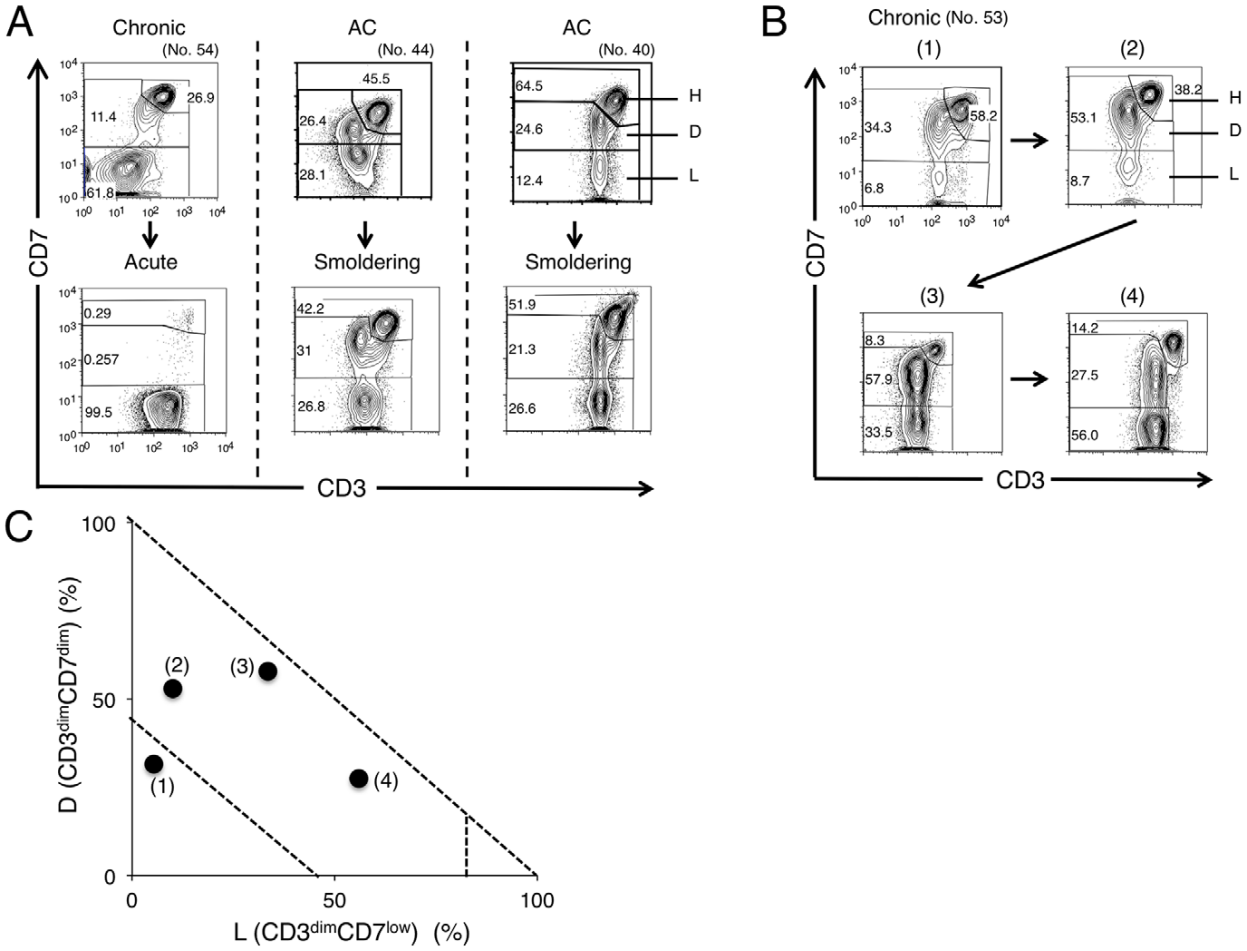
Alteration in the CD3 versus CD7 profile by flow cytometry in accordance with disease progression. (A) Change in the CD3 versus CD7 profile in representative cases. In all three cases shown, change in clinical data (*e.g.*, abnormal lymphocyte, LDH) resulted in progression of the clinical subtype. (B) Change in the CD3 versus CD7 profile in a time course in the case of chronic-type ATL. Clinical data are shown in Table S1. (C) Flow cytometric data in (B) are summarized in the D(%) versus L(%) plot.

In several cases, we could obtain time-sequential samples (Figure 4). The patient (no. 54) shown on the left in Figure 4A progressed from chronic-type to acute-type disease. In flow cytometric analysis, decreases in the H and D subpopulations and an increase in the L subpopulation were observed, indicating that disease progression correlated well with the change in the CD3 versus CD7 profile. The patients in the middle (no. 44) and on the right (no. 40) were included in Group 2 at the AC stage and later progressed to smoldering-type ATL. Although variation in the change of the flow cytometric profile was seen between these patients, the results suggest that ACs in Group 2 are at high risk of developing ATL.

The patient in Figure 4B (no. 53) was initially diagnosed with AC and later progressed to chronic-type ATL. Although the initial clinical course was stable, an increase in abnormal lymphocyte numbers was later observed, and low-dose VP-16 therapy (50 mg/day) was initiated because of hypoxemia due to lung infiltration of ATL cells. Table S1 and Figure 4C show summaries of the clinical data and the flow cytometric analyses, respectively. The flow cytometric data correlated well with disease progression.

## Discussion

Findings in our previous analysis of acute-type ATL samples prompted our analysis of various clinical subtypes of patients infected with HTLV-I to examine whether the CD3 versus CD7 profile reflects the progression of oncogenesis in HTLV-I-infected cells [17]. Representative flow cytometric data shown in Figure 1A suggested that the CD3 versus CD7 profile changed during disease progression. As the disease stage progressed, the D and L subpopulations increased with concomitant decreases in the CD3^high^CD7^high^(H) subpopulation. Figure 1C, a summary of the flow cytometric data of all cases analyzed, reveals that the two-dimensional plot of the proportions of the D versus L subpopulations could divide all cases into three groups. Group 1, the area under the diagonal line, equivalent to 55% of the H subpopulation in which all normal controls were included (Figure S2), contained the majority of HTLV-I ACs. Group 3 was the area beyond 80% of the L subpopulation, and the majority of acute-type cases were included in this group. Group 2, located between Groups 1 and 3 (*i.e.*, less than 55% of the H subpopulation and 80% of the L subpopulation), included indolent-type (smoldering and chronic) cases and some AC cases. These results suggest that the CD3 versus CD7 expression profile reflects disease stage. Initially, both the D and L subpopulations gradually and simultaneously increased. However, at the clinically advanced stage, the increase in the L subpopulation was prominent. The change is considered to reflect the biological difference between the D and L subpopulations, which needs to be clarified.

In HTLV-I infection, the small clones of infected cells are considered to coexist from the AC stage [19], [20]. A selected clone from the multiple small clones then grows and progresses to the malignant state, and the emergence of a dominant clone indicates disease progression in ATL [19], [20]. As shown in Figure 2B–D, major bands suggesting dominant clones were evident in patients with progressed clinical subtypes or those in the advanced group in the CD3 versus CD7 profile, and major bands existed exclusively in the D and L subpopulations. These data also support the idea that increases in the D and L subpopulations correlate with the progression of disease stage. AC cases in Group 2 had high HTLV-I proviral loads (>4%; Figure 1B) and clear major bands were observed by inverse long PCR in these cases (Figure 2B, right). Sasaki *et al.* reported that two cases of HTVL-I AC with oligoclonal bands on Southern blots and high VLs (20%) had progressed to ATL by 4 and 3.5 years later [21]. The two cases may correspond to HTLV-I AC in Group 2 proposed in our study. In fact, two cases of ACs in our series that were included in Group 2 progressed to smoldering ATL (Figure 4A). AC cases in Group 2 could be regarded as advanced carriers. Our flow cytometric analysis could apparently discriminate high-risk AC cases from stable ones. Follow-up analysis of these cases is warranted to determine whether AC cases included in Group 2 progress to ATL. Flow cytometric data for these AC cases included in Group 2 (Figure 1A and 1C) were similar to those for indolent ATL cases in Group 2. These ACs in Group 2 can be considered essentially the same as smoldering ATL cases. Some of the ACs categorized according to Shimoyama's criteria should in fact be separated and regarded as a subtype together with at least some of the smoldering ATL cases.

Iwanaga *et al.* reported that high HTLV-I proviral load (>4%) in whole PBMCs was a risk factor for progression to ATL [13]. In Figure 1B, the ACs with VLs>4% were distributed between Groups 1 and 2. These findings suggest that not all ACs with high VLs are currently in an advanced stage, although they may have the potential to develop ATL in the future.

In general, the categorization by flow cytometric profile correlated well with the current classification of clinical subtypes, with some exceptional cases of acute-type and smoldering-type disease (Figure 3). The only manifestation of three smoldering cases categorized in Group 1 was skin lesions; they fell into Group 1 because they showed minimal abnormalities in peripheral blood [22]. Three acute-type ATL cases categorized in Group 2 had indolent clinical courses. A diagnosis of acute-type disease is made when the indolent-type and lymphoma-type are excluded, according to Shimoyama's criteria. The CD3 versus CD7 plot may discriminate the cases that will follow an indolent clinical course from the aggressive acute-type ATL.

The VL in each subpopulation indicated that HTLV-I-infected cells were relatively concentrated in the D and L subpopulations (representative data are shown in Figure 2A). These data are consistent with downregulation of CD3 and CD7 being relevant to HTLV-I infection, although cells without HTLV-I infection may also contribute to this change to some extent, as a substantial subpopulation of T cells has been reported to be CD7-deficient under physiological [23], [24] and certain pathological conditions, including autoimmune disorders and viral infection [25]–[29]. To more precisely analyze phenotypic changes in HTLV-I-infected cells, markers that indicate HTLV-I infection should be incorporated in future studies.

**Figure 5:**
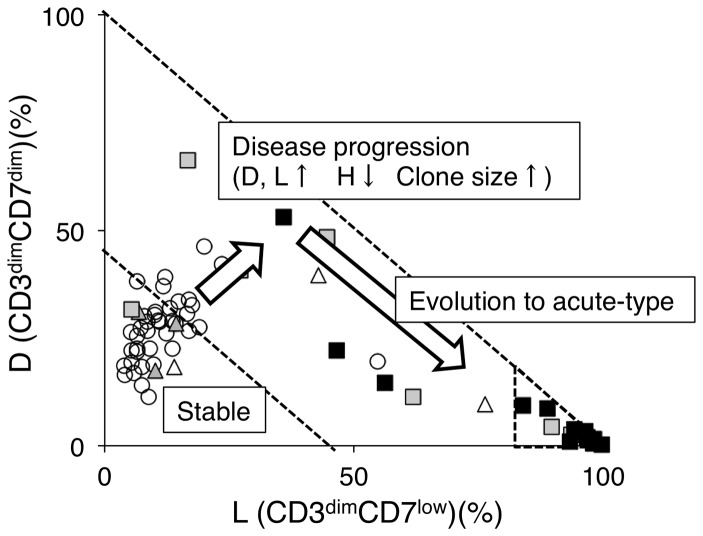
Summary of the study: the CD3 versus CD7 profile reflects progression of disease stage in patients infected with HTLV-I. In the percentage of D (CD3^dim^CD7^dim^) versus L (CD3^dim^CD7^low^) plot, Group 1 includes the majority of AC cases. As disease stage progresses, the CD3 versus CD7 profile then changes. With downregulation of CD3 and CD7, the D and L subpopulations increase gradually (Group 2). During this step, clones in the D and L subpopulations increase in size. Further accumulation of genetic alterations will result in rapid expansion of ATL clones—*i.e.*, evolution to acute-type ATL. In this step, the CD3 versus CD7 profile will progress from Group 2 to 3.

A summary of this study is shown in Figure 5. In the CD3 versus CD7 profile, most AC cases were included in Group 1, in which the D and L subpopulations were relatively small. Consistent with disease progression to smoldering- and chronic-type ATL, a decrease in the H subpopulation and increases in the D and L subpopulations occur (Group 2). In this step, increases in the sizes of clones in the D and L subpopulations are observed. Further expansion of the leukemic clone results in progression to acute-type ATL in which the L subpopulation has expanded (Group 3). According to a study by Yamaguchi *et al.*, the natural course of ATL is to progress from the HTLV-I carrier state through the intermediate state, smoldering ATL, and chronic ATL, and finally to the acute ATL, indicating a process of multistage leukemogenesis [19]. We consider this study to successfully link the progressive clinical status and phenotypic changes in HTLV-I-infected cells. However, the way in which this profile reflects multistep oncogenesis in HTVL-I infection at the molecular level remains unclear. Further molecular analyses of the three subpopulations will help in understanding the mechanism(s).

## Supporting Information

Figure S1 **Representative flow cytometric analysis of an HTLV-I asymptomatic carrier (patient no. 32).** The CD3 versus CD7 plot of CD4^+^ cells was constructed according to the gating procedure shown in this figure. In the plot, we designated three subpopulations: H (CD3^high^CD7^high^), D (CD3^dim^CD7^dim^), and L (CD3^dim^CD7^low^).
(PPTX)

Figure S2 **A two-dimensional plot of 10 normal controls showing the percentage of the D and L subpopulations.**
(PPTX)

Figure S3 **Southern blot analysis of clonal integration of the HTLV-I provirus.** Representative data (AC, No. 34) are shown. In *Eco*RI or *Pst*I digestion, a band indicated by a red arrow represents the monoclonal integration of the provirus. The band pattern indicates that two major clones coexist. This analysis was performed by a commercial laboratory (SRL, Tokyo, Japan).
(PPTX)

Table S1 **Clinical data in a case of chronic-type ATL (No. 53).** Proportion of abonormal lymphocytes in the preipheral blood WBC were evaluated by morphological exmanination. LDH: Lactate dehydrogenase (normal range, 120–240 U/L) sIL-2R: soluble interleukin-2 receptor (normal range, 122–496 U/ml).
(XLSX)

## References

[1] Yoshida M, Miyoshi I, Hinuma Y (1982) Isolation and characterization of retrovirus from cell lines of human adult T-cell leukemia and its implication in the disease. Proc Natl Acad Sci U S A 79: 2031–2035. 6979048 10.1073/pnas.79.6.2031 PMC346116

[2] Osame M, Usuku K, Izumo S, Ijichi N, Amitani H, et al (1986) HTLV-I associated myelopathy, a new clinical entity. Lancet 1: 1031–1032. 10.1016/s0140-6736(86)91298-5 2871307

[3] Mochizuki M, Watanabe T, Yamaguchi K, Takatsuki K, Yoshimura K, et al (1992) HTLV-I uveitis: a distinct clinical entity caused by HTLV-I. Japanese journal of cancer research : Gann 83: 236–239. 1582883 10.1111/j.1349-7006.1992.tb00092.x PMC5918816

[4] Proietti FA, Carneiro-Proietti AB, Catalan-Soares BC, Murphy EL (2005) Global epidemiology of HTLV-I infection and associated diseases. Oncogene 24: 6058–6068. 16155612 10.1038/sj.onc.1208968

[5] Yamaguchi K, Watanabe T (2002) Human T lymphotropic virus type-I and adult T-cell leukemia in Japan. International journal of hematology 76 Suppl 2: 240–245. 12430931 10.1007/BF03165123

[6] Murphy EL, Hanchard B, Figueroa JP, Gibbs WN, Lofters WS, et al (1989) Modelling the risk of adult T-cell leukemia/lymphoma in persons infected with human T-lymphotropic virus type I. International journal of cancer Journal international du cancer 43: 250–253. 2917802 10.1002/ijc.2910430214

[7] Shimoyama M (1991) Diagnostic criteria and classification of clinical subtypes of adult T-cell leukaemia-lymphoma. A report from the Lymphoma Study Group (1984–87). Br J Haematol 79: 428–437. 1751370 10.1111/j.1365-2141.1991.tb08051.x

[8] Tsukasaki K, Hermine O, Bazarbachi A, Ratner L, Ramos JC, et al (2009) Definition, prognostic factors, treatment, and response criteria of adult T-cell leukemia-lymphoma: a proposal from an international consensus meeting. J Clin Oncol 27: 453–459. 19064971 10.1200/JCO.2008.18.2428 PMC2737379

[9] Takasaki Y, Iwanaga M, Imaizumi Y, Tawara M, Joh T, et al (2010) Long-term study of indolent adult T-cell leukemia-lymphoma. Blood 115: 4337–4343. 20348391 10.1182/blood-2009-09-242347

[10] Hisada M, Okayama A, Shioiri S, Spiegelman DL, Stuver SO, et al (1998) Risk factors for adult T-cell leukemia among carriers of human T-lymphotropic virus type I. Blood 92: 3557–3561. 9808547

[11] Imaizumi Y, Iwanaga M, Tsukasaki K, Hata T, Tomonaga M, et al (2005) Natural course of HTLV-1 carriers with monoclonal proliferation of T lymphocytes (“pre-ATL”) in a 20-year follow-up study. Blood 105: 903–904. 15632212 10.1182/blood-2004-06-2489

[12] Kamihira S, Atogami S, Sohda H, Momita S, Yamada Y, et al (1994) Significance of soluble interleukin-2 receptor levels for evaluation of the progression of adult T-cell leukemia. Cancer 73: 2753–2758. 8194016 10.1002/1097-0142(19940601)73:11<2753::aid-cncr2820731117>3.0.co;2-x

[13] Iwanaga M, Watanabe T, Utsunomiya A, Okayama A, Uchimaru K, et al (2010) Human T-cell leukemia virus type I (HTLV-1) proviral load and disease progression in asymptomatic HTLV-1 carriers: a nationwide prospective study in Japan. Blood 116: 1211–1219. 20448111 10.1182/blood-2009-12-257410

[14] Okamoto T, Ohno Y, Tsugane S, Watanabe S, Shimoyama M, et al (1989) Multi-step carcinogenesis model for adult T-cell leukemia. Japanese journal of cancer research : Gann 80: 191–195. 2498254 10.1111/j.1349-7006.1989.tb02289.x PMC5917708

[15] Matsuoka M, Jeang KT (2007) Human T-cell leukaemia virus type 1 (HTLV-1) infectivity and cellular transformation. Nat Rev Cancer 7: 270–280. 17384582 10.1038/nrc2111

[16] Yoshida M (2010) Molecular approach to human leukemia: isolation and characterization of the first human retrovirus HTLV-1 and its impact on tumorigenesis in adult T-cell leukemia. Proceedings of the Japan Academy Series B, Physical and biological sciences 86: 117–130. 10.2183/pjab.86.117 PMC3417562 20154469

[17] Tian Y, Kobayashi S, Ohno N, Isobe M, Tsuda M, et al (2011) Leukemic T cells are specifically enriched in a unique CD3(dim) CD7(low) subpopulation of CD4(+) T cells in acute-type adult T-cell leukemia. Cancer science 102: 569–577. 21205081 10.1111/j.1349-7006.2010.01833.x

[18] Sugamura K, Fujii M, Kannagi M, Sakitani M, Takeuchi M, et al (1984) Cell surface phenotypes and expression of viral antigens of various human cell lines carrying human T-cell leukemia virus. International journal of cancer Journal international du cancer 34: 221–228. 6088403 10.1002/ijc.2910340213

[19] Yamaguchi K, Kiyokawa T, Nakada K, Yul LS, Asou N, et al (1988) Polyclonal integration of HTLV-I proviral DNA in lymphocytes from HTLV-I seropositive individuals: an intermediate state between the healthy carrier state and smouldering ATL. British journal of haematology 68: 169–174. 2894835 10.1111/j.1365-2141.1988.tb06185.x

[20] Mortreux F, Gabet AS, Wattel E (2003) Molecular and cellular aspects of HTLV-1 associated leukemogenesis in vivo. Leukemia : official journal of the Leukemia Society of America, Leukemia Research Fund, UK 17: 26–38. 10.1038/sj.leu.2402777 12529656

[21] Sasaki D, Doi Y, Hasegawa H, Yanagihara K, Tsukasaki K, et al (2010) High human T cell leukemia virus type-1(HTLV-1) provirus load in patients with HTLV-1 carriers complicated with HTLV-1-unrelated disorders. Virology journal 7: 81. 20423527 10.1186/1743-422X-7-81 PMC2876101

[22] Setoyama M, Katahira Y, Kanzaki T (1999) Clinicopathologic analysis of 124 cases of adult T-cell leukemia/lymphoma with cutaneous manifestations: the smouldering type with skin manifestations has a poorer prognosis than previously thought. The Journal of dermatology 26: 785–790. 10659498 10.1111/j.1346-8138.1999.tb02093.x

[23] Reinhold U, Abken H (1997) CD4+ CD7- T cells: a separate subpopulation of memory T cells? J Clin Immunol 17: 265–271. 9258765 10.1023/a:1027318530127

[24] Reinhold U, Abken H, Kukel S, Moll M, Muller R, et al (1993) CD7- T cells represent a subset of normal human blood lymphocytes. J Immunol 150: 2081–2089. 7679701

[25] Aandahl EM, Quigley MF, Moretto WJ, Moll M, Gonzalez VD, et al (2004) Expansion of CD7(low) and CD7(negative) CD8 T-cell effector subsets in HIV-1 infection: correlation with antigenic load and reversion by antiretroviral treatment. Blood 104: 3672–3678. 15308569 10.1182/blood-2004-07-2540

[26] Autran B, Legac E, Blanc C, Debre P (1995) A Th0/Th2-like function of CD4+CD7- T helper cells from normal donors and HIV-infected patients. J Immunol 154: 1408–1417. 7529803

[27] Legac E, Autran B, Merle-Beral H, Katlama C, Debre P (1992) CD4+CD7-CD57+ T cells: a new T-lymphocyte subset expanded during human immunodeficiency virus infection. Blood 79: 1746–1753. 1373086

[28] Schmidt D, Goronzy JJ, Weyand CM (1996) CD4+ CD7- CD28- T cells are expanded in rheumatoid arthritis and are characterized by autoreactivity. J Clin Invest 97: 2027–2037. 8621791 10.1172/JCI118638 PMC507276

[29] Willard-Gallo KE, Van de Keere F, Kettmann R (1990) A specific defect in CD3 gamma-chain gene transcription results in loss of T-cell receptor/CD3 expression late after human immunodeficiency virus infection of a CD4+ T-cell line. Proc Natl Acad Sci U S A 87: 6713–6717. 2144349 10.1073/pnas.87.17.6713 PMC54607

