# Supplementary figures and images for: The CD3 versus CD7 Plot in Multicolor Flow Cytometry Reflects Progression of Disease Stage in Patients Infected with HTLV-I
Source: PLoS One. 2013 Jan 22;8(1):e53728. doi: 10.1371/journal.pone.0053728 (PMC3551918; doi:10.1371/journal.pone.0053728)

## Slide 1
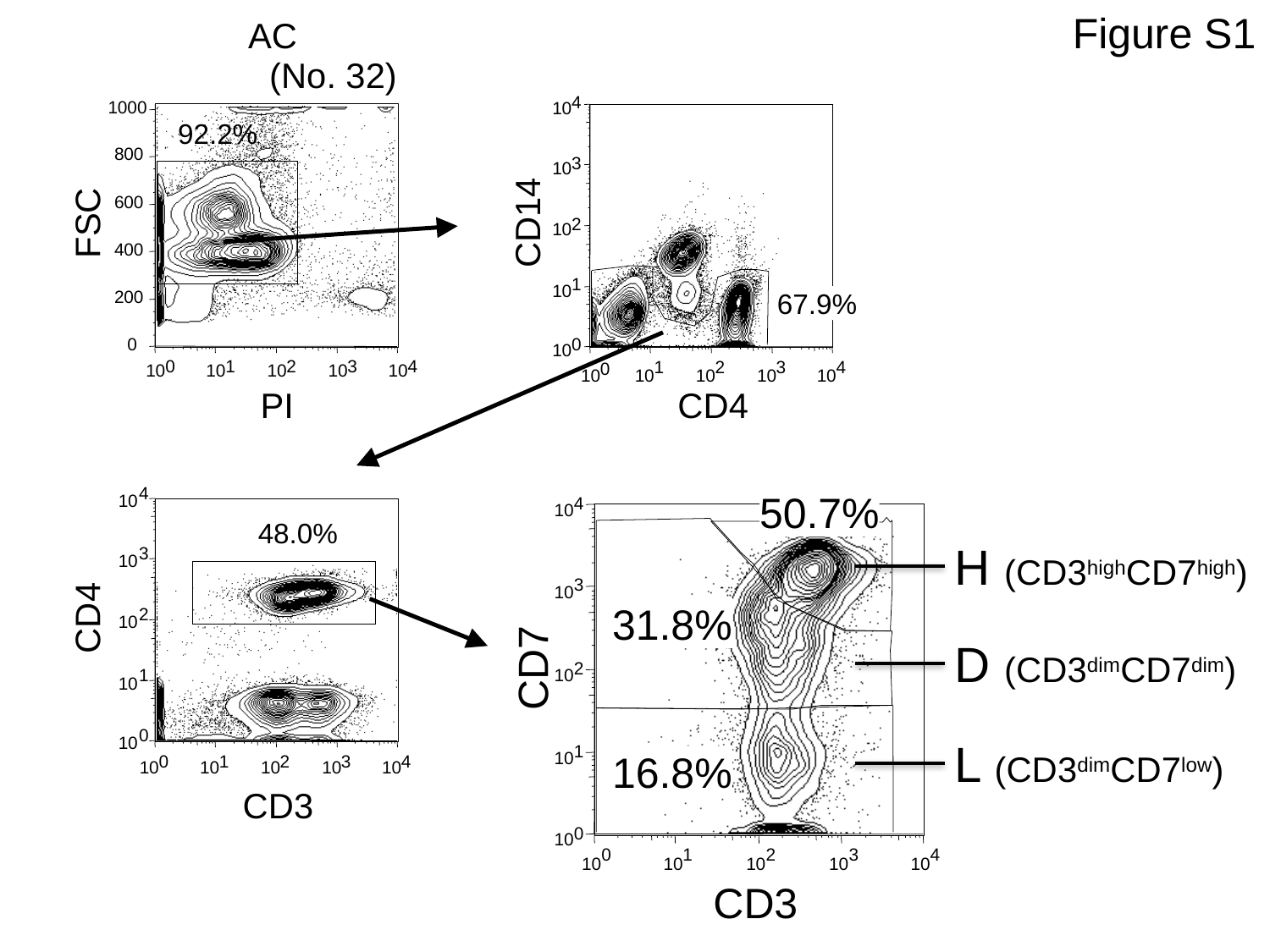

Figure S1
AC
(No. 32)
4
1000
10
92.2%
800
3
10
600
CD14
FSC
2
10
400
1
10
200
67.9%
0
0
10
0
1
2
3
4
1
2
3
4
0
10
10
10
10
10
10
10
10
10
10
PI
CD4
4
50.7%
10
4
10
48.0%
H (CD3highCD7high)
3
10
3
10
CD4
31.8%
2
10
D (CD3dimCD7dim)
CD7
2
1
10
10
0
10
L (CD3dimCD7low)
1
16.8%
10
0
1
2
3
4
10
10
10
10
10
CD3
0
10
0
1
2
3
4
10
10
10
10
10
CD3

Supplement: Figure S1 — Representative flow cytometric analysis of an HTLV-I asymptomatic carrier (patient no. 32). The CD3 versus CD7 plot of CD4+ cells was constructed according to the gating procedure shown in this figure. In the plot, we designated three subpopulations: H (CD3highCD7high), D (CD3dimCD7dim), and L (CD3dimCD7low). (PPTX) [file pone.0053728.s001.pptx]

## Slide 1
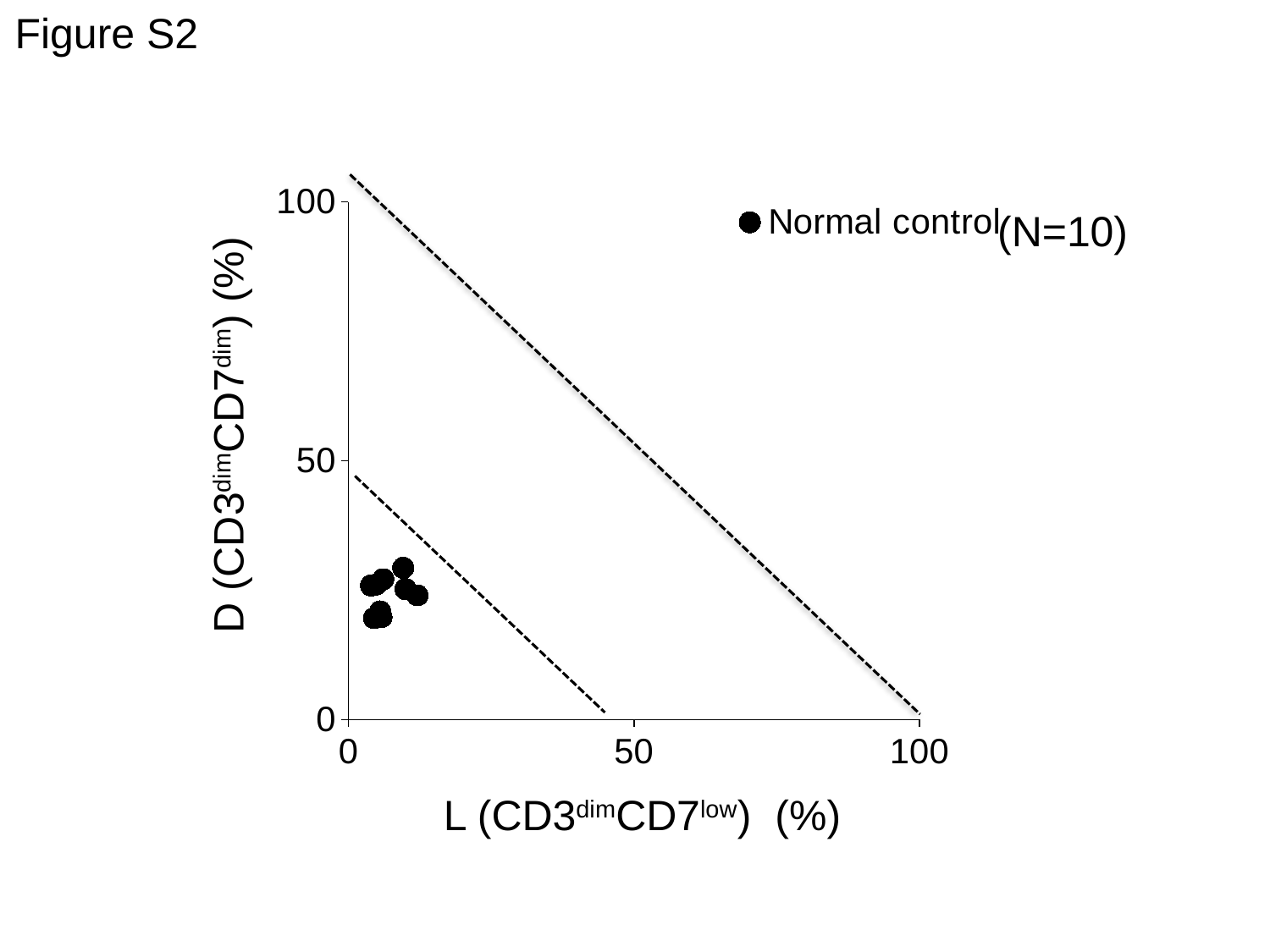

Figure S2
### Chart:
| Category | |
|---|---|(N=10)
D (CD3dimCD7dim) (%)
L (CD3dimCD7low) (%)

Supplement: Figure S2 — A two-dimensional plot of 10 normal controls showing the percentage of the D and L subpopulations. (PPTX) [file pone.0053728.s002.pptx]
