# Supplementary material for: The CD3 versus CD7 Plot in Multicolor Flow Cytometry Reflects Progression of Disease Stage in Patients Infected with HTLV-I
Source: PLoS One. 2013 Jan 22;8(1):e53728. doi: 10.1371/journal.pone.0053728 (PMC3551918; doi:10.1371/journal.pone.0053728)
Supplement: Figure S3 — Southern blot analysis of clonal integration of the HTLV-I provirus. Representative data (AC, No. 34) are shown. In EcoRI or PstI digestion, a band indicated by a red arrow represents the monoclonal integration of the provirus. The band pattern indicates that two major clones coexist. This analysis was performed by a commercial laboratory (SRL, Tokyo, Japan). (PPTX) [file pone.0053728.s003.pptx]

## Slide 1
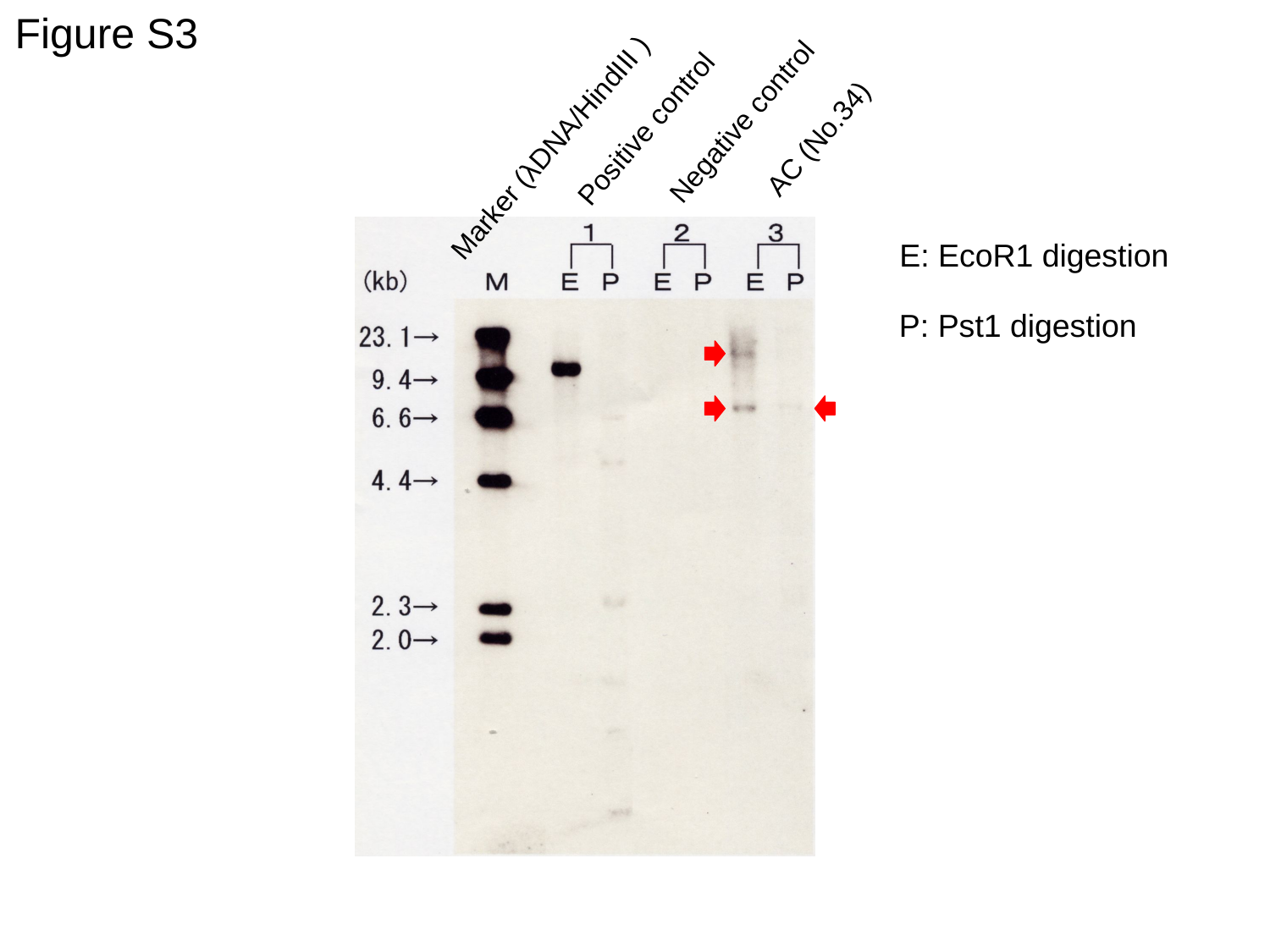

Figure S3
Negative control
Positive control
AC (No.34)
Marker (λDNA/HindIII )
E: EcoR1 digestion
P: Pst1 digestion
